# Optimization of Intraoperative Neural Monitoring of the Recurrent Laryngeal Nerve in Thyroid Surgery

**DOI:** 10.3390/medicina58040495

**Published:** 2022-03-30

**Authors:** Chia-Yuan Hsieh, Hao Tan, Hui-Fang Huang, Tzu-Yen Huang, Che-Wei Wu, Pi-Ying Chang, David-Vi Lu, I-Cheng Lu

**Affiliations:** 1 Department of Anesthesiology, Kaohsiung Municipal Siaogang Hospital, Kaohsiung Medical University, Kaohsiung 807, Taiwan; kobethechampion81@gmail.com (C.-Y.H.); zealotjacky@gmail.com (H.T.); wiebittee@gmail.com (H.-F.H.); 2 Department of Otorhinolaryngology—Head and Neck Surgery, Kaohsiung Medical University Hospital, Kaohsiung Medical University, Kaohsiung 807, Taiwan; tyhuang.ent@gmail.com (T.-Y.H.); cwwu@kmu.edu.tw (C.-W.W.); 3 Faculty of Medicine, College of Medicine, Kaohsiung Medical University, Kaohsiung 807, Taiwan; 4 Department of Anesthesiology, Kaohsiung Municipal Ta-Tung Hospital, Kaohsiung Medical University, Kaohsiung 807, Taiwan; annabelle69@gmail.com

**Keywords:** thyroid surgery, recurrent laryngeal nerve, intraoperative neural monitoring, surface electrodes, neuromuscular block

## Abstract

The application of intraoperative neural monitoring (IONM) has been widely accepted to improve surgical outcomes after thyroid surgery. The malfunction of an IONM system might interfere with surgical procedures. Thus, the development of anesthesia modalities aimed at ensuring functional neuromonitoring is essential. Two key issues should be taken into consideration for anesthetic management. Firstly, most patients undergo recurrent laryngeal nerve monitoring via surface electrodes embedded in an endotracheal tube. Thus, advanced video-assisted devices might optimize surface electrode positioning for improved neuromonitoring signaling accuracy. Secondly, neuromuscular blocking agents are routinely used during thyroid surgery. The ideal neuromuscular block should be deep enough for surgical relaxation at excision and recovered enough for an adequate signal f nerve stimulation. Proper neuromuscular block management could be achieved by titration doses of muscle relaxants and reversal agents.

## 1. Introduction

Thyroid surgery is globally a high-volume surgery with approximately 150,000 thyroidectomies performed per year in the U.S. [1]. In our healthcare system, the number of thyroid surgeries has nearly doubled compared with the past decade. Nowadays, thyroid surgery is safe with a low complication rate. A recurrent laryngeal nerve (RLN) or external branches of superior laryngeal nerve (EBSLN) injury remains an unwanted complication of thyroid surgery. Intraoperative neural monitoring (IONM) of the RLN has obtained a growing acceptance as a standard adjuvant for thyroid surgery [2,3,4,5,6,7,8,9,10,11,12]. In a meta-analysis of 1513 thyroidectomy patients, the temporary RLN palsy rate was 4.2% with neural monitoring and 7.7% without it. The permanent RLN palsy rate with and without neural monitoring was 1.0% and 1.6%, respectively [13]. The ultimate goal of IONM during thyroid surgery is to minimize the risk of recurrent or superior laryngeal nerve injury. Moreover, the reported benefits of an IONM system during thyroid surgery include: (1) the identification of the RLN or vagus nerve; (2) the real-time monitoring of target nerve integrity; and (3) the evaluation of the nerve injury mechanism [14,15,16].

A functional IONM system is based on the precise position of the electromyography (EMG) endotracheal tube and adequate recovery from a neuromuscular block. Most recurrent laryngeal nerve monitoring during thyroid surgery is achieved via surface electrodes incorporated into an EMG tube. The initial step of successful IONM is to avoid/correct the malposition of an EMG tube. The pros and cons of intubation devices have been summarized from conventional direct laryngoscopies [8,17] to video-assisted intubation devices [18,19]. Secondly, neuromuscular blocking agents are considered to be a mandatory part of general anesthesia to facilitate tracheal intubation and surgical relaxation. The extent of the neuromuscular block degree (NMB) is also critical to evoke EMG signals of the target nerves. Proper NMB management through the timing and dosage of reversal agents such as sugammadex and neostigmine are undergoing increasing amounts of investigations [20]. This comprehensive review aims to optimize intraoperative neural monitoring of the recurrent laryngeal nerve in thyroid surgery by accumulating the clinical research.

## 2. Placement of Endotracheal Tube with Surface Electrodes

A typical EMG endotracheal tube provides both a patient airway for ventilation and electrodes that directly contact the vocal cords. The surface electrodes may be embedded exposed wire electrodes (Xomed NIM), conductive silver ink electrodes (NIM Trivantage), or adhesive stick-on electrodes (Neurovision Dragonfly, NIM FLEX) [21,22]. According to the learning curve for IONM in thyroid surgery, most technical problems resulted from an improper depth or alignment of the electrodes [23].

Successful tracheal intubation to ensure an airway and ventilation is a precondition for every general anesthetic. A precise surface electrode position on the vocal cords is a precondition for functional neuromonitoring. Patient positioning is an important element of intubation preparation. When a conventional laryngoscope is used, maintaining the head and neck at a neutral or sniff position is suggested [8]. When a video laryngoscope is chosen, successful intubation could be fulfilled for thyroid surgery with a neck extension with a shoulder roll or donut head pad [24]. Moreover, a neck extension during intubation meets the patient position need and may prevent the alteration of the tube position [8]. An ideal intubation device should meet both demands. Each device should be assessed by a successful tracheal intubation and precise electrode positioning. This review summarizes the available intubation devices to place an EMG tube and confirms the electrode positioning (Table 1).

### 2.1. Direct Laryngoscope

Direct laryngoscopy with a Macintosh blade is used to distract the upper airway and allow a direct visualization of the larynx with an intact field of view and without image distortion from indirect lenses [29,30]. Direct laryngoscopy remains a standard and valuable skill for tracheal intubation in various scenarios, including the emergency department, intensive care units, and operation rooms. Although direct laryngoscopy is a mandatory technique for all physicians, difficult intubation is inevitable and common among the general population, occurring in 1.8–5.8% of cases [31]. This incidence was noted to be higher among patients undergoing thyroid surgery, occurring in 5.3–24.6% of cases [11,12,13].

With respect to the placement of an EMG tube for a monitored thyroidectomy, the successful rate on the first attempt via direct laryngoscopy was as high as 94.3–96.4% in experienced anesthesiologists [17,25]. In a 4-year period of clinical reviews, 2.3% (8/336) of patients required a second method to accomplish the intubation task [25].

The limitations of this conventional intubation device include a higher intubation difficulty scale, longer intubation, and lower success rate compared with a video-assisted laryngoscope or an intubating stylet in both normal and difficult intubation scenarios [25,32,33].

### 2.2. Fiber-Optic Bronchoscope

A flexible fiber-optic bronchoscope (FOB) allows for the direct visualization of both the upper and lower airways. It is a valuable device for the diagnosis or treatment of pulmonary disease and the establishment of a secure airway [34,35]. The two roles of a FOB in IONM include: (1) its use as an alternative management for difficult intubation or in awake or anesthetized patients that cannot be intubated [25,36]; and (2) its use as an examination to confirm the accurate surface electrode positions of an EMG tube [8]. The examination or adjustment of the EMG tube position can be assisted by inserting a FOB via a nasal route.

For intubation, Chang et al. reported a total of 8 “cannot intubate” events in 336 direct laryngoscopy procedures; 3 of the 8 patients underwent successful intubation by FOB with a second attempt [25]. Anguraj demonstrated a successful fiber-optic nasal intubation in an anticipated difficult airway resulting from thyroid cancer with a tracheal invasion [36]. Fiber-optic intubation is not recommended as a routine practice because a FOB via the oral route encounters a higher difficulty and placing an EMG tube via the nasal route may cause trauma concerns.

For examinations, a FOB provided an adjustment of the EMG tube position for displacement only in 3.6–5.7% of patients [8,17]. A FOB is effective but time-consuming to check the tube position; thus, we believe a fiber-optic examination can be waived in most conditions with cumulative experience and a modified protocol [37,38].

### 2.3. Video-Optical Intubating Stylet

The optical stylet is an intubation device with optical fibers inside a metal or plastic tube. It was initially introduced to clinical intubation in 1979. However, the original products did not gain commercial success due to unfriendly practices and significant experience demand [39]. The new generation of video-optical intubating stylets obtained clinical popularity and commercial success after 2010. They usually consist of a rigid or semi-rigid stylet, a light source, video chips instead of optic fibers, and a monitor screen attached to the handle [40]. Video-optical intubating stylets have been shown to be effective for tracheal intubation compared with conventional laryngoscopes and video laryngoscopes.

With respect to tracheal intubation during a monitored thyroidectomy, the use of the Trachway intubating stylet (Biotronic Instruments Enterprise Ltd., Taichung, Taiwan) in 412 patients depicted a 99% (408/412) successful intubation rate on the first attempt. The study concluded that the intubating stylet was better than conventional laryngoscopy with a higher success rate and lower difficulty of intubation [25]. Liu et al. used the Shikani optical stylet in 40 patients with difficult airways due to thyroid tumors and showed that 90% (36/40) of patients were intubated successfully on the first attempt [26].

The major disadvantage of the intubating stylet is its limited visual field. With respect to the surface electrode position, it only allows for visualization via the inner lumen of an EMG tube. The surface electrodes on the outer side of an EMG tube cannot be observed by this method. Fortunately, a proper EMG tube depth according to a reference value can be suitable in most circumstances [17]. A widely used EMG tube (Trivantage; Medtronic Xomed Inc., Jacksonville, FL, USA) has been designed to have a longer electrode working length (49 mm) to detect EMG. The Trivantage tube has a higher range to tolerate displacement or rotation compared with an EMG tube with a 30 mm working length [17,41].

### 2.4. Video Laryngoscope

A video laryngoscope is a laryngoscope equipped with a video camera to indirectly visualize the glottis structure; hence, the conventional view of direct laryngoscopy through the oral pharyngeal to laryngeal axis was no longer mandatory. This indirect laryngoscopy has improved the visualization of the glottis, decreased the lifting force to the tongue, and reduced cervical movement compared with direct laryngoscopy [42,43].

The advantages of video laryngoscopy in placing an EMG tube include a high intubation success rate, the confirmation of the electrode positions, and the adjustment of a malposition if necessary [18,24,27,28]. In an EMG tube placement protocol, a UESCOPE video laryngoscope (UE Medical Devices, Inc., Newton, MA, USA) was utilized to achieve 100% (40/40) successful intubation in patients undergoing a monitored thyroidectomy (Table 1). All of the patients showed functional IONM and only one patient (2.5%) needed a further EMG tube position adjustment [24].

Although video laryngoscopy is useful and feasible in most scenarios for EMG tube placement, it has a few disadvantages. First, the blade is difficult to insert in a patient with very poor dentures or a severely limited mouth opening. Second, the camera image might be obscured by secretions, blood, or exhaled air. Finally, expertise is required to obtain a good view, manipulate the EMG tube, and prepare for a possible difficult intubation [44].

## 3. Neuromuscular Blocking Agents

Muscle relaxation, analgesia, and hypnosis are the triad of general anesthesia. The utilization of neuromuscular blocking agents facilitate tracheal intubation and surgical relaxation. Once tracheal intubation is complete, the degree of neuromuscular block turns into a key factor for EMG signaling during thyroid surgery with IONM [45,46]. The ideal neuromuscular block profile includes a maximum at tracheal intubation, is light enough when an EMG signal is required, and is deep enough to avoid unwanted movements during the entire operation. Hence, the use of neuromuscular blocking agents for IONM should take onset, duration, and dose titration into consideration. To optimize IONM signaling, the duration of a neuromuscular blocking agent and the time interval between its administration and obtaining the EMG signal play key roles. Commonly available NMBAs are summarized below.

### 3.1. No Neuromuscular Blocking Agents

The use of no neuromuscular blocking agent is not a recommended regimen for a routine monitored thyroidectomy. The only advantage of abandoning neuromuscular blocking agents is to avoid the influence of muscle relaxation on the EMG signals from the target nerves. However, the disadvantages of this regimen may be significant. First, from an anesthetic perspective, it does not provide sufficient intubation conditions in most patients and leads to greater intubation-related airway trauma [47,48]. Second, from a surgical perspective, inadequate surgical relaxation might also result in a higher anesthetic and analgesic consumption as well as more intraoperative limb movements or bucking events.

### 3.2. Short Duration: Suxamethonium (Succinylcholine)

Suxamethonium is a regional preference for routine neural monitoring anesthesia in many institutes because of its unique advantage. However, it should be used with caution when considering its risk/benefit balance and other options of feasible neuromuscular blocking agents (i.e., rocuronium, cisatracurium) [20]. The unique advantage of suxamethonium for EMG monitoring is its pharmacological properties. Suxamethonium, also known as succinylcholine, is a depolarizing neuromuscular blocking agent with a rapid onset of 60 s and ultra-short duration within 5–10 min. A single intubation dose of suxamethonium provides sufficient relaxation for a tracheal intubation and the timely restoration of neuromuscular transmissions for monitoring nerve integrity [15]. Although suxamethonium possesses an ideal pharmacological profile for neural monitoring, it may be associated with various adverse events from minor (such as myalgia and dysrhythmia) to fatal (such as hyperkalemia and malignant hyperthermia) [49].

### 3.3. Intermediate Duration: Rocuronium and Aminosteroid Agents

Rocuronium has been highly recommended as a mainstay of neural monitoring anesthesia [38,50,51,52]. Both rocuronium and vercuronium are commonly used agents with an aminosteroid structure. Three advantages of rocuronium for a monitored thyroidectomy are a rapid onset, titratable duration, and reversal by a selective binding agent, namely, sugammadex. Regarding the onset, rocuronium in 0.83 and 1.04 mg/kg doses has reported a 90% and 95% probability of successful intubation within 60 s, respectively [53]. The rapid onset time is not inferior to succinylcholine. For duration, rocuronium in 0.83 and 1.04 mg/kg doses showed a 32 and 46 min neuromuscular block duration, respectively [53]. To meet the monitored thyroidectomy demand, it is feasible to titrate the rocuronium dose to shorten the duration of the neuromuscular block. For example, rocuronium at 0.3 and 0.6 mg/kg at anesthesia induction showed a different duration and detectable EMG signals in 100% and 53% of patients at initial vagal stimulation, respectively [52]. Regarding reversal, only rocuronium has a specific antagonist, sugammadex, to effectively restore neuromuscular function whenever obtaining an EMG signal is necessary [20]. The disadvantages of rocuronium include its high dependency on the liver metabolism and its ability to cause hypersensitivity [54,55].

### 3.4. Intermediate Duration: Cisatracurium and Isoquinoline Agents

In recent randomized control trials, cisatracurium at 1.6 or 2 times of the 95% effective dose was recommended as a cost-effective alternative to the rocuronium–sugammadex protocol [56,57]. When two effective doses (0.1 mg/kg) were administrated, the time to a detectable EMG signal was 32 min [57] and the average initial EMG amplitude was 448 μV [56]. Cisatracurium is a commonly used non-depolarizing agent with an isoquinoline structure. The outstanding advantages of cisatracurium in anesthesia include a non-histamine release as with other isoquinoline agents (i.e., atracurium) as well as metabolism by Hoffman elimination independent of liver and renal functions. The pharmacological characteristics are feasible not only for the general population but also for critical illness and geriatric patients [58,59]. There are two minor drawbacks of cisatracurium for neural monitoring during thyroid surgery. First, in low doses (one effective dose at 0.05 mg/kg), a more difficult laryngoscopy and higher intubation difficulty were noted compared with two effective doses [56,57]. Second, in regular doses (two effective doses at 0.1 mg/kg), the duration of the neuromuscular block lasts 45–50 min without a specific reversal agent as with the rocuronium–sugammadex protocol [60].

## 4. Neuromuscular Blockade Reversal

“To reverse or not to reverse, that is the question”. The neuromuscular block degree is one of the most important parameters for successful IONM during clinical surgery. Too deep a neuromuscular block degree will diminish the EMG amplitude; too light a degree may be associated with unwanted movements or bucking. The proper management of the neuromuscular block can assure excellent tracheal intubation conditions, adequate surgical relaxation, and the timely restoration of IONM signaling. The development stages of neuromuscular block management to obtain timely IONM signals are listed below.

### 4.1. Pre-Sugammadex Era

Before the introduction of sugammadex into the reversal of a rocuronium-induced neuromuscular block, a spontaneous recovery via the titration of the NMBA dose was the only way to manage the neuromuscular block degree. A full dose of any NMBA (i.e., rocuronium or atracurium) was feasible for IONM but a delayed or suppressed IONM signal occurred in a few cases [50,51]. During this era, a titration of rocuronium to one effective dose (0.3 mg/kg) was considered as an alternative option to enable high-quality IONM signaling in all patients at the expense of suboptimal intubating conditions in a small portion of patients [52].

### 4.2. Sugammadex Era

The primary goal of implementing sugammadex into the anesthesia practice was to prevent postoperative residual curarisation or a residual neuromuscular block after extubation during the postoperative care [61,62]. Sugammadex is a modified c-cyclodextrin produced to reverse aminosteroid NMBAs (mainly rocuronium and vecuronium) by encapsulating them to form a complex without a neuromuscular blocking action. It has been reported that sugammadex not only ensures the effective reversal of the neuromuscular block but also reduces postoperative pulmonary complications (i.e., respiratory failure and pneumonia) [63,64]. The intraoperative reversal of a neuromuscular block by sugammadex can restore timely EMG signaling when the evoked target nerve is mandatory for a thyroidectomy. Table 2 summarizes the recent clinical trials investigating the effect of reversal agents on IONM during thyroid surgery [65,66,67,68,69,70,71].

With similar high success rates for IONM, a decline was noted in the effective sugammadex dose from the first literature report in 2016. A dose of 2 mg/kg in reference to extubation was effective for high-quality IONM signaling at any surgical step. When an IONM conductive system was set up effectively, sugammadex could be used as a routine protocol to guarantee a high EMG amplitude or rescue undetectable EMG signaling [65,66,67]. However, this dose was associated with unwanted patient movements or bucking in as high as 20–35% of cases [65,66,67,68,69]. In recent reports, a dose of 1.0 or even 0.5 mg/kg was also sufficient to ensure a high-quality EMG signal in all patients with preserved surgical relaxation [69,70].

### 4.3. Neostigmine Returns

With respect to neuromuscular reversal for postoperative recovery, sugammadex is undoubtedly superior to neostigmine. However, little is known about neostigmine reversal for IONM [71]. Traditionally, neostigmine is used to reverse broad-spectrum NMBAs; either isoquinoline NMBAs (e.g., atracurium and cisatracurium) or steroidal NMBAs (e.g., rocuronium and vecuronium) [72].

Recently, Oh et al. demonstrated a novel use of neostigmine reversal for IONM during thyroid surgery. The study showed that neostigmine (2 mg)–glycopyrrolate (0.4 mg) was a cost-effective option to allow for sufficient IONM signaling with minimal bucking events in 4% (2/50) of patients [71]. The combination of neostigmine and anticholinergics (e.g., glycopyrrolate or atropine) was a standard regimen to prevent bradycardia events. With respect to neuromuscular reversal for IONM during thyroid surgery, neostigmine may not be inferior to sugammadex. Neostigmine deserves further investigation in its timing, dosing, and risk/benefit issues.

## 5. Conclusions

To optimize IONM for recurrent laryngeal nerves in thyroid surgery, advanced video-assisted intubation devices are recommended to fulfill a successful tracheal intubation and to confirm the proper positioning of an EMG endotracheal tube. The titration of any intermediate-effect NMBA could be feasible for IONM during thyroid surgery. Both intraoperative sugammadex and neostigmine have demonstrated a sufficient neuromuscular reversal for a high-quality EMG amplitude. The combination of rocuronium and sugammadex has been considered as a standard to reverse a neuromuscular block at any degree and restore EMG signaling at any time. A further investigation of neostigmine and its role in neuromuscular reversal is required.

## Figures and Tables

**Table 1:** Rating for intubation devices to place an endotracheal tube and confirm electrode positioning.

Intubation Devices		Successful Tracheal Intubation	Precise Surface Electrode Position
Macintosh laryngoscope [8,17]	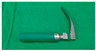	+	+
Fiber-optic bronchoscope [8,17]	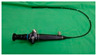	N/A ^1^	++
Video-optical intubating stylet [25,26]	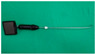	++	N/A ^2^
Video laryngoscope [24,27,28]	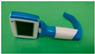	++	++

^1^ Not applicable for routine tracheal intubation as an alternative for difficult intubation; ^2^ not applicable for observing electrode positions; +: good; ++: excellent.

**Table 2:** Clinical trials investigating the effect of reversal agents on IONM during thyroid surgery.

Authors and Published Year	Patients (*n*)	Groups (*n*)	Reversal Agent/Dose	Timing of Reversal/ NMBA ^1^: Reversal Interval	IONM Outcomes/ Mean V1 ^2^ Amplitude (μV)
Lu et al., 2016 [65]	50	1	Sugammadex 2 mg/kg	Skin incision 16 min	100% V1: 1202 ± 563 µV
Kontoudi et al., 2016 [66]	75	3	Sugammadex 2 mg/kg	Not mentioned 15 min	96% good quality V1 Not mentioned
De Vendin et al., 2017 [67]	120	1	Sugammadex not mentioned	If V1 < 100 μV 40 min	Rescue for no IONM signal Not mentioned
Gune et al., 2019 [68]	129	2	Sugammadex 2 mg/kg	At V0 24 min	100% V1: 567 ± 219
Chai et al., 2021 [69]	102	2	Sugammadex 2 or 1 mg/kg	Tube fixation <5 min	100% V1: 1086 ± 673 vs. 1162 ± 728
Lu et al., 2021 [70]	80	2	Sugammadex 0.5 mg/kg	10 min after skin incision 26 min	100% V1: 1214 ± 6231
Oh et al., 2021 [71]	50	1	Neostigmine 2 mg	Tube fixation <5 min	100% V1: 985 ± 472

^1^ NMBA: neuromuscular blocking agents; ^2^ V1: vagus nerve stimulation before thyroid dissection.
